# Immune function differences between two color morphs of the red palm weevil *Rhynchophorus ferrugineus* (Coleoptera: Curculionidae) at different life stages

**DOI:** 10.1002/ece3.7474

**Published:** 2021-03-31

**Authors:** Guihua Wang, Yuxuan Zhou, Baozhen Tang, Habib Ali, Youming Hou

**Affiliations:** ^1^ State Key Laboratory of Ecological Pest Control for Fujian and Taiwan Crops Fujian Agriculture and Forestry University Fuzhou China; ^2^ Key Laboratory of Biopesticide and Chemical Biology Ministry of Education Fujian China; ^3^ Fujian Province Key Laboratory of Insect Ecology College of Plant Protection Fujian Agriculture and Forestry University Fujian China; ^4^ Department of Agriculture Engineering Khawaja Fareed University of Engineering and Informtion Technology Rahim Yar Khan Pakistan; ^5^ University of Agriculture Faisalabd Okara Pakistan

**Keywords:** antimicrobial activity, cuticle pigmentation, hemocyte, immune response, melanism, pathogen resistance, phenoloxidase

## Abstract

Several studies demonstrated that in insects cuticle melanism is interrelated with pathogen resistance, as melanin‐based coloration and innate immunity possess similar physiological pathways. For some insects, higher pathogen resistance was observed in darker individuals than in individuals with lighter cuticular coloration. Here, we investigated the difference in immune response between two color morphs (black and red) and between the life stages (pupa and adult) of the red palm weevil *Rhynchophorus ferrugineus* (Coleoptera: Curculionidae). Here in this study, cuticle thickness, microbial test (antimicrobial activity, phenoloxidase activity, and hemocyte density), and immune‐related gene expression were evaluated at different stages of RPW. Study results revealed that cuticle thickness of black phenotype was thicker than red phenotype at old‐pupa stage, while no significant difference found at adult stage. These results may relate to the development processes of epidermis in different stages of RPW. The results of antimicrobial activity, phenoloxidase (PO) activity, and hemocyte density analyses showed that adults with a red phenotype had stronger pathogen resistance than those with a black phenotype. In addition to antimicrobial activity and PO activity, we tested relative gene expression in the fat body of old pupae. The results of hemolymph antimicrobial analysis showed that old pupae with a red phenotype were significantly different from those with a black phenotype at 12 hr after *Staphylococcus aureus* injection, suggesting that red phenotype pupae were more sensitive to *S. aureus*. Examination of gene expression in the fat body also revealed that the red phenotype had a higher immune response than the black phenotype. Our results were inconsistent with the previous conclusion that dark insects had increased immune function, suggesting that the relationship between cuticle pigmentation and immune function in insects was not a direct link. Additional possible factors that are associated with the immune response, such as life‐history, developmental, physiological factors also need to be considered.

## Graphical abstract

This paper reported pathogen resistance differences between two different segmental ventral colorations in the red palm weevil Rhynchophorus ferrugineus (Coleoptera: Curculionidae) (red phenotype and black phenotype) and investigated immune function in the old‐pupa and adult stages. Our results showed cuticle melanism pigmentation does not correspond to enhanced immune function in RPW, which were inconsistent with the previous conclusion that dark insects had increased immune function. We suggest that evaluation of the relationship between cuticle pigmentation and immune function in insects needs to also consider additional possible factors that are associated with the immune response, such as life‐history, developmental, and physiological factors.

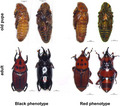

## 1 INTRODUCTION

Cuticle pigmentation is widespread in the animal kingdom, including invertebrates and vertebrates, and different species have specific pigment patterns (Costin & Hearing, 2007). Pigmentation patterns are one of the most distinguishable features of insect morphology and have attracted a great attention of scientists and researchers. Insect pigmentation provided a useful foundation for the study of evolutionary development biological theory, phenotype genetics, and physiology. Cuticle pigmentation varies not only across species but also in different development stages in the same species.

Body pigmentation of insects varies according to season transitions and environmental changes (Futahashi & Fujiwara, 2008). Cuticle pigmentation diversity is one of the most conspicuous evidence of biodiversity in nature and is involved with a series of physiological processes, such as camouflage, warning coloration, and behavior (Hill, 1992; Kettlewell, 1973; Majerus, 1998). For insects, body coloration often confers substantial fitness benefits, such as thermoregulation, food acquisition, mate recognition, and protection from being preyed on by predators (Badejo et al., 2020; Caro et al., 2017; Krams et al., 2016; Talloen et al., 2004; True, 2003).

Insect pigmentation requires the proper functioning of physiological processes that involve a variety of enzymes. Several types of chemical pigments have been reported in different insect species, such as melanins, pterins, anthraquinones, ommochromes, aphins, tertapyrroles, carotenoids, and flavonoids/anthocyanins (Shamim et al., 2014). Pigments and their precursors are typically synthesized in epidermal cells and transported into the epithelial layer via hemolymph of insects (Ashida & Brey, 1995). A study of the Asian ladybird beetle *Harmonia axyridis* showed that carotenoid content is responsible for the variable orange‐red coloration of elytra, and this red hue appears to be correlated with the content of their defensive alkaloid molecules (Bezzerides et al., 2007). In addition, melanin production is a common phenomenon in insects. The common cuticle tanning process begins with tyrosine and forms a series of pigments, including melanin‐like pigment, NADA‐pigment (N‐acetyldopamine‐pigment), and NBAD‐pigment (N‐β‐alanyldopamine‐pigment) (Noh et al., 2016). Studies suggested that the melanin‐like and quinonoid pigments produced by tyrosine metabolism play a major role in the darkening of beetle cuticle, such as the red flour beetle *Tribolium castaneum* (Arakane et al., 2009; Gorman & Arakane, 2010; Noh et al., 2015). There is a hypothesis that melanin‐producing insects have advantages in environmental adaptation, especially immune defense. Thicker epidermis presented by darker individuals compared with lighter ones (Evison et al., 2017), suggesting physical body protection. Melanin pigments and their precursors also play an immunological body protective role against parasites (Griffith et al., 2006; Marmaras et al., 1996; Nappi & Vass, 1993), which creates a direct link between pigmentation and pathogen resistance (Wilson et al., 2001). Phenoloxidases (POs) have been reported extensively in many insects that participate in the synthesis of cuticle melanin and wound healing and are also important in the innate immune response (Andersen, 2005; Cerenius et al., 2008; Cerenius & Söderhäll, 2004; Kanost et al., 2004; Söderhäll & Cerenius, 1998).

The red palm weevil (RPW), *Rhynchophorus ferrugineus* (Coleoptera: Curculionidae), is one of the most devastating invasive palm pests in tropical and subtropical areas (Abbas, 2010; Faleiro, 2006). Color trait in this pest includes red‐brown and black phenotypes, which is observed on ventral side in the old‐pupa and adult stages. Gregarious life habit, wood‐boring behavior, and widespread host ranges of RPW require that they must have high pathogen resistance that is vital in the adaptive environment of insects. The insect development process was accompanied by many physiological changes. Pigmentation was an accumulated process from old‐pupa to adult stages of RPW, which may reveal a life‐history trade‐off relationship. This study aimed to investigate the difference in immune response between two color morphs of RPW at different life stages. We speculated that black weevils had higher pathogen resistance than red weevils. We mainly investigated hemocyte counts (hemocyte numbers/density), antibacterial activity, and PO activity to compare the immunity between the two color lines of RPW.

## 2 MATERIALS AND METHODS

### 2.1 Insect cultures

**FIGURE 1:**
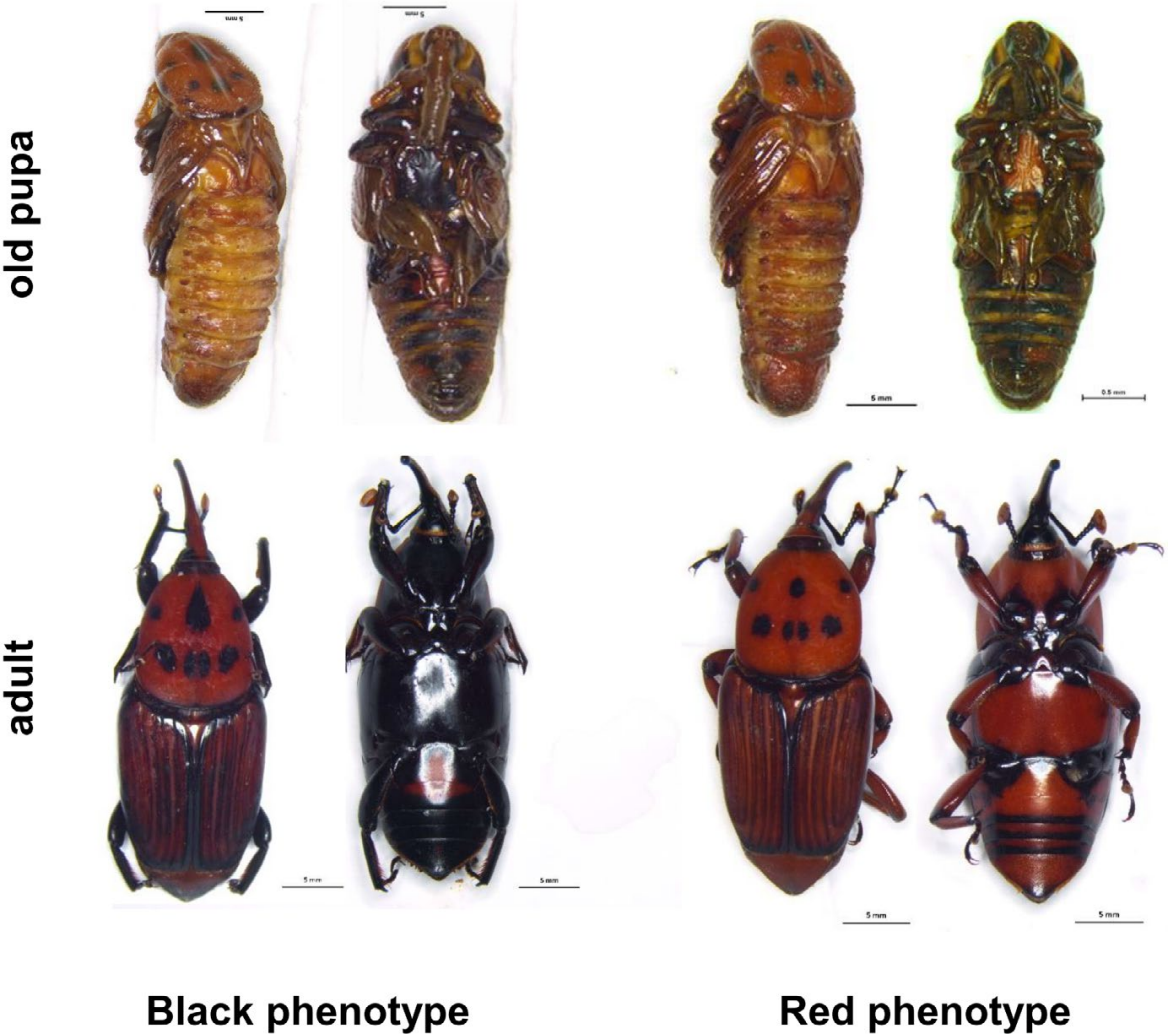
Dorsal and ventral views of old pupa and adult of *Rhynchophorus ferrugineus* with two color phenotypes

Cuticular color lines were selected on the ventral side of beetles (black and red‐brown) (Figure 1). RPW color lines were collected from the field and raised under laboratory conditions (Temperature: 25 ± 1°C, Humidity: 75%, LD: 0:24 hr for larvae; LD: 12:12 hr for adults). Pupa of RPW can be divided into three phases (lasting about 15 days, including prepupa, nonpigment pupa, and old pupa). We chose old pupae (average 8‐days pupa, stage in which pigmentation occurred) and adults for further treatment. The population size of black phenotype and red phenotype raised in laboratory was 927 and 759 individuals, respectively.

### 2.2 Cuticle thickness

Cuticle thickness of metathorax was tested by toluidine blue staining. Metathorax cuticle of old pupae and 7‐day‐old adults was dissected carefully by forceps and scissors in PBS buffer and stored in 2.5% glutaric dialdehyde for 6 hr. Samples were rinsed three times by 0.1 M sodium cacodylate buffer for 15 min. And then dehydrated in an ethanol gradient of 50%, 70%, 80%, and 90% for 15–20 min each at 4°C. Samples were infiltrated in acetone and resin (3:1) for 3–4 hr, 1:1 acetone: resin treated overnight, 1:3 acetone: resin for 3–4 hr at room temperature and then resin treated 3 times each for 2–3 hr at 37°C. Samples were dry at 37°C overnight, and 45°C for 12 hr, 60°C for 24 hr followed by Semi‐thin sectioning. Semi‐thin sections were stained with toluidine blue staining for 10 min and imaged under the microscope. The cuticle thickness was measured by NIS‐N‐viewer software (Nikon Ni‐U). Three semi‐thin sections with 10 digital images per sample from each color phenotype at old pupa and adult were selected, respectively.

### 2.3 Antimicrobial assay

A hole in the head of pupae or neck of adults was made with medical needles, then used 10 μl pipettor to collect the flow out hemolymph, and quickly stored in an ice centrifuge tube for further experiment. A total of 60 μl hemolymph per sample was collected from old pupae or adults of both color lines. Each color line collected 4–5 biological replications, each replication (sample) contained 3 old pupae or 5 adults. Each sample contained 60 μl of (phenylthiourea, 5 mM) PTU, which prevented hemolymph melanization, and was diluted with 360 μl of Schneider Insect Medium (Sigma‐Aldrich, S9895). Diluted hemolymph was centrifuged at 6,500 *g* for 10 min at 4°C. The supernatant was filtered by a 0.22‐μm cellulose acetate filter (Merck Millipore Ltd.) according to Fang et al. (2016) to remove the bacterial in hemolymph. The antimicrobial activity of different color line weevils was tested by turbidimetry according to Shi et al. (2014), and a larger OD_600_ value change suggested lower antimicrobial activity. A volume of 30 μl of insect medium instead of diluted hemolymph supernatant was used as a negative control for each well, while tetracycline (20 μg/ml) was used as a positive control. Each of the 96‐well plate systems included 30 μl of hemolymph supernatant and 60 μl of *Escherichia coli* or *Staphylococcus aureus* bacterial solution (37°C, shaking at 200 rpm overnight, and 1,000 times dilution, final concentration 10^5^). OD_600_ data were collected at 0, 4, 6, 8, 12, and 24 hr.

### 2.4 Bacterial challenge

Old pupae and 7‐day‐old adults of both color lines were collected for a bacterial challenge assay. The insects were separated into three groups. The first group was used as the untreated group. The second group was injected with 2 μl of *E. coli* (OD_600_ was 1.9, Wang, 2018) per insect, and another group was treated with 2 μl of phosphate‐buffered saline (PBS) as a control.

#### 2.4.1 Basal PO activity assay

L‐dopa is the substrate of PO, which can be used to quantify basal PO. This enzymatic reaction results in a darkening in the test solution from the production of dopachrome, and the change in the absorbance of the solution can be quantified with a spectrophotometer at 490 nm. The greater the rate of conversion of L‐dopa to dopamine is, the higher the activity of basal PO.

Hemolymphs were collected from the bacterial challenge group (the second group) and the PBS injection group (the third group) at 0, 6, 12, and 24 hr post‐treatment. Five microliters of hemolymph per beetle was directly saved in liquid nitrogen and then stored at −80°C for further assays. Every sample consisted of 3 beetles, with 4 replications for each color line.

PO activity assay of RPW was revised according to Shi et al. (2014). Hemolymph was diluted with ice‐cold PBS by 1:10 and centrifuged at 8,000 *g* for 10 min at 4°C, and the supernatant was collected. A 150 μl cell‐free hemolymph was used; a sterile 96‐well plate culture cluster (COSTAR ref # 42592, Corning Incorporated) was placed on ice, and 30 μl of PBS (10 mM sodium phosphate) was added to each of the 96 wells. Twenty microliters of each sample (hemolymph supernatant) and a volume of 100 μl of L‐DOPA buffer (4 mg/ml) were added to each well. The reaction was allowed to proceed at 30°C in a spectrophotometer for 30 min and the absorbance was read every minute at 490 nm. Basal PO enzyme activity was measured as the slope of the reaction curve during the linear phase of the reaction.

#### 2.4.2 Testing the circulating hemocyte counts (CHCs)

Five microliters and ten microliters of hemolymph per beetle were collected from adults and old pupa of the untreated group (the first group) and 12 hr post‐bacterial and PBS treatments (the second and third groups) and diluted with 10 μl of ice‐cold PBS in a 0.5 ml centrifuge tube, while the old‐pupa hemolymph did not need to dilute and directly used for counting. The solution was vortexed and pipetted onto a hemocytometer with Neubauer chamber (1 mm × 1 mm, 25 × 16 grid, Qiujing, Shanghai) for hemocyte counting. The total cell counts in five large grids were used to assess the hemocyte density. Thirty individuals were collected for each treatment.

### 2.5 Relative gene expression in old pupae

The total hemocyte counts are too low to detect mRNA abundance in the old pupa, thus we analyzed transcripts of immunity‐related genes, including two antimicrobial peptides (attacin and cecropin) and two prophenoloxidase (PPO) genes (*CL4037‐2* and *unigene15235*), in the fat body tissues. Insect treatments were according to 2.3 bacterial challenge under three groups. Fat body tissues were dissected carefully by forceps and scissors in PBS, every three individuals as one replication, every line contained 3–5 replications. To compare the difference of transcriptional levels of the above genes in old pupae between the two color lines, template cDNA was prepared from total RNA isolated from the fat body of old pupae using TRIzol reagent (Invitrogen) according to the manufacturer's protocol. A TransScript All‐in‐One First‐strand cDNA synthesis kit (TransGen Biotech) was used for cDNA synthesis. A real‐time PCR was conducted in a final volume of 20 μl containing 2 μl of the template cDNA, 0.4 μl of each primer (10 mM), 10 μl of FastStart Universal SYBR Green Master (ROX) (Roche), and 7.2 μl of ddH_2_O, with the following program: initial denaturation at 95°C for 10 min, followed by 40 cycles of 95°C for 15 s and 60°C for 1 min. GAPDH was used to normalize differences in the concentration of cDNA templates among samples. The primers used for qPCR are listed in Table S1.

### 2.6 Statistical analysis

OD_600_ values at 0, 4, 6, 12, and 24 hr for antimicrobial activity were collected, and ∆OD_600_ values at 12 and 24 hr for *E*. *coli* and *S*. *aureus* treatments were analyzed between the two color lines. We defined a 0.001 OD_490_ value change per min as 1 U, and relative PO activity (*E*. *coli* injection groups relative to the mean value of the PBS injection group) was used to measure the difference between the two color lines. Hemocyte density was detected by the following formula: hemocyte density/ml = (sum of five grid counts)/80 × 400 × 10^4^ × dilution ratio. Considering the originate hemocyte density may different between two color phenotypes without treatment, the change of hemocyte counts before and after injection (Relative CHC_PBS‐12 hr_ or Relative CHC*_E. coli_*_‐12 hr_ = CHC_PBS‐12 hr_/CHC_untreated‐mean_ or CHC*_E. coli_*_‐12 hr_/CHC_untreated‐mean_) was measured. The 2^−∆∆CT^ value was used to assess relative gene expression. SPSS 17.0 (SPSS Inc.) was used for statistical analysis, and all the charts were made in GraphPad Prism 7.0 (GraphPad Software). Except for the antimicrobial peptides gene expression used ANOVA analysis, *t* test was used to all other results between two color lines.

## 3 RESULTS

### 3.1 Cuticle thickness

**FIGURE 2:**
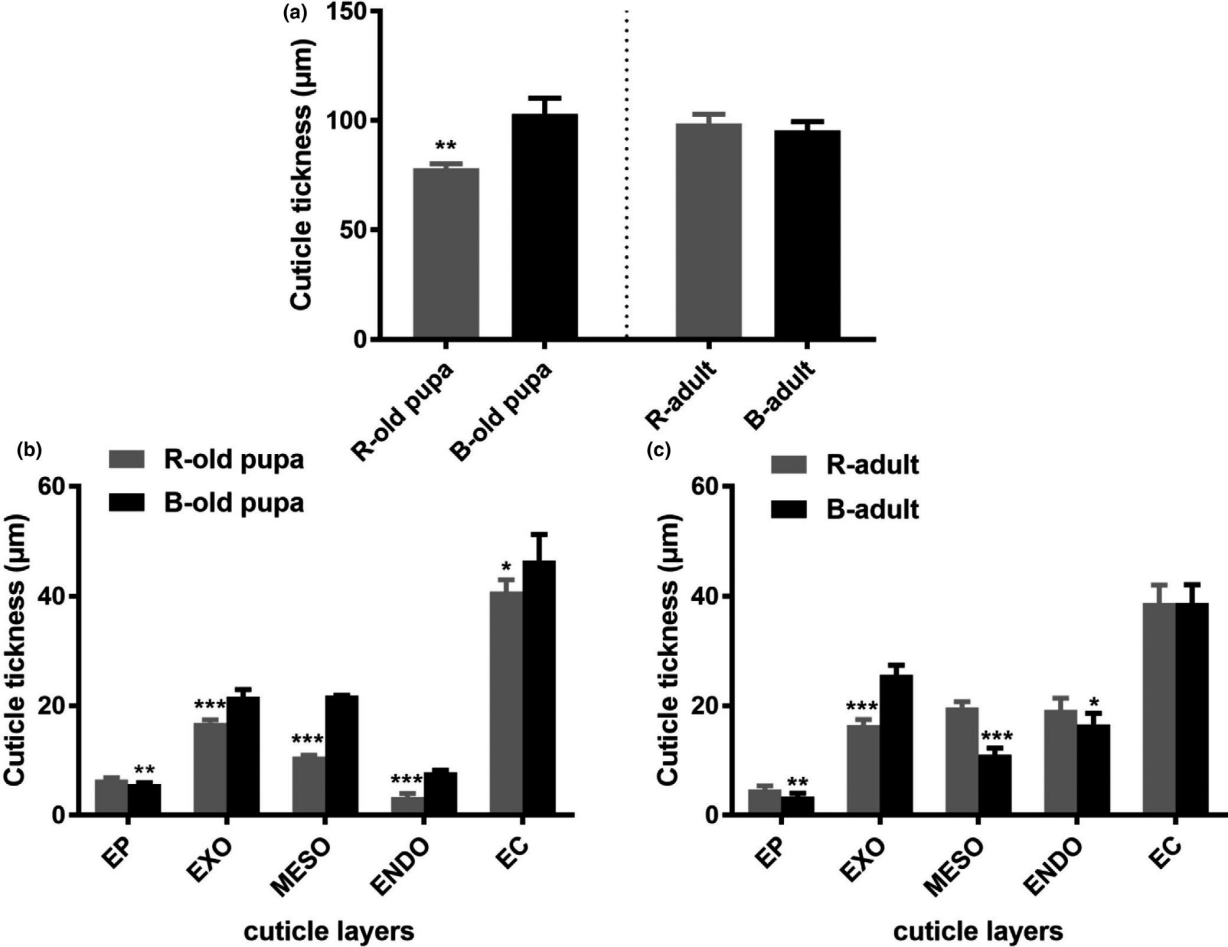
Cuticle thickness between two color lines of RPW at old‐pupa and adult stages. (a) the whole cuticle thickness in old pupa and adult; (b) thickness in different cuticle layers in old pupa; (c) thickness of different cuticle layers in adult. EP, epicuticle; EXO, exocuticle; MESO, mesocuticle; ENDO, endocuticle; EC, epidermal cell. *, ** and *** mean significant difference at the levels of *p* < .05, .01 and .001 by *t* test, respectively

The structure of cuticle included five levels as follows: epicuticle (EP), exocuticle (EXO), mesocuticle (MESO), endocuticle (ENDO), and epidermal cell (EC). The whole cuticle thickness of black phenotype was significant thicker than red phenotype at old pupa (*t*_9_ = −8.606, *p* < .001), while no significant difference found at adult stage (*t*_20_ = 1.597, *p* = .126) (Figure 2a). The thickness of MESO and ENDO in red phenotype was rapidly increased during old pupa (MESO: *t*_8_ = −68.540, *p* < .001; ENDO: *t*_9_ = −11.882, *p* < .001) (Figure 2b) to adult stage (MESO: *t*_20_ = 15.781, *p* < .001; ENDO: *t*_20_ = 2.444, *p* = .024) (Figure 2c).

### 3.2 Antimicrobial activity

**FIGURE 3:**
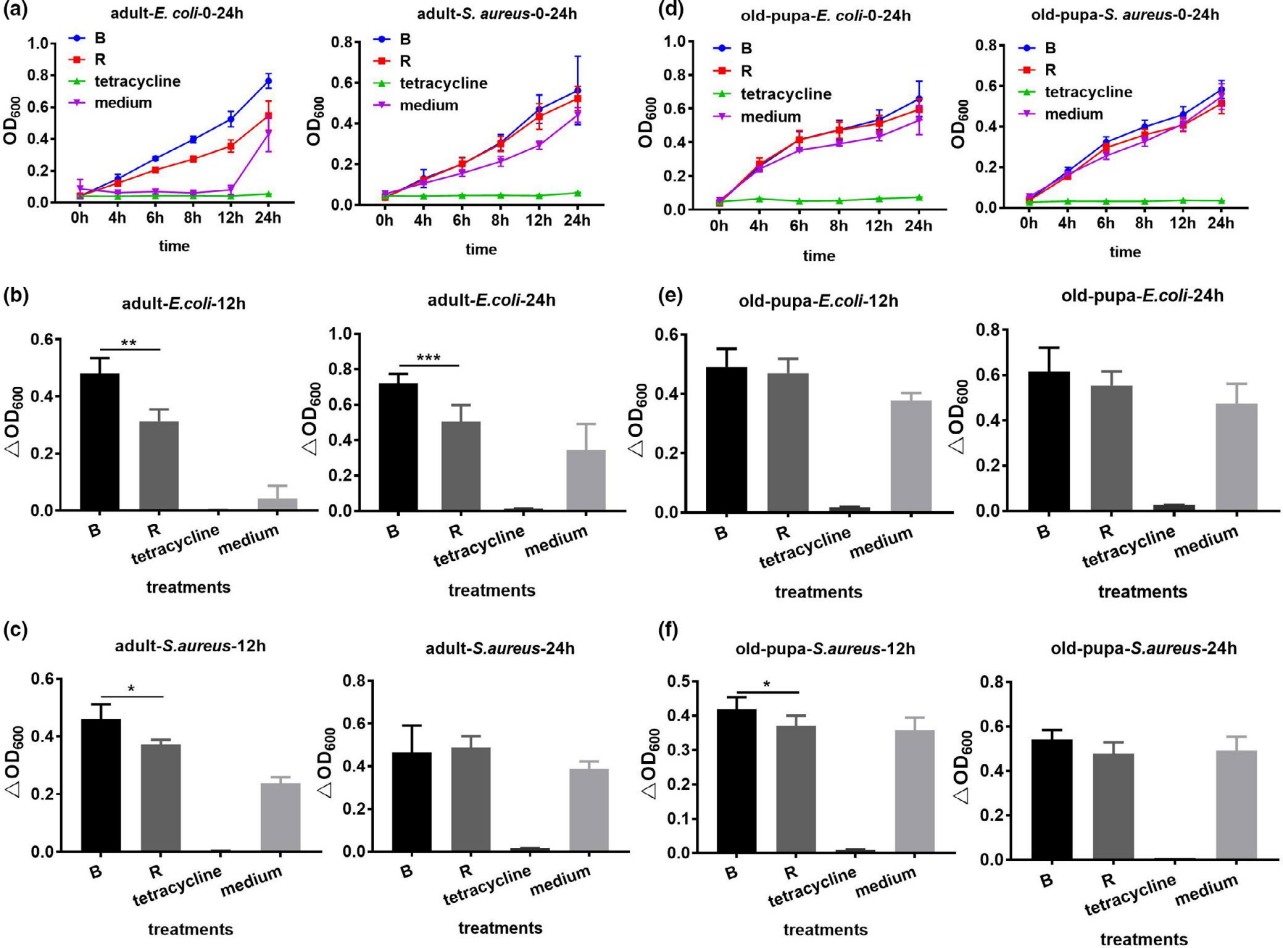
Antimicrobial activity of the two phenotypes after bacterial challenge in old pupa and adult of RPW. (a,d) the tendency of OD_600_ value across different time points in adult and old pupa stages, respectively, after *Escherichia coli* and *Staphylococcus aureus* infection. (b,c) antimicrobial activity in adult stage at 12 and 24 hr after *E. coli* and *S. aureus* challenge, respectively. (e,f) antimicrobial activity in old pupae stage at 12 and 24 hr after *E. coli* and *S. aureus* challenge, respectively. *, ** and *** mean significant difference at the levels of *p* < .05, .01 and .001 by *t* test, respectively

Based on the dynamic tendency of the hemolymph of the two color lines against microbial challenge, similar tendencies were found between the two color lines of adults and pupae after different bacterial treatments (Figure 3a,d). The lines of treatment groups above control groups showed that hemolymph of RPW may contain some substance that could promote the growth of bacterial comparing with insect medium as the negative control. The difference of antimicrobial activity between two color lines was mainly considered although the antimicrobial activity of hemolymph in RPW was lower than insect medium. Relative antimicrobial activity results showed that adults of the red phenotype exhibited significantly higher pathogen resistance than those of the black phenotype 12 hr (*t*_6_ = 5.002, *p* = .002) and 24 hr (*t*_6_ = 26.133, *p* < .001) post‐*E*. *coli* treatment and 12 hr after *S. aureus* challenge (*t*_6_ = 2.815, *p* = .048) (Figure 3b,c). However, in old pupae, a significant difference was found only at 12 hr (*t*_8_ = 2.332, *p* = .048) after *S. aureus* treatment, which also suggested that the red phenotype of old pupae had enhanced resistance (Figure 3e,f).

### 3.3 Basal PO activity and hemocyte density

**FIGURE 4:**
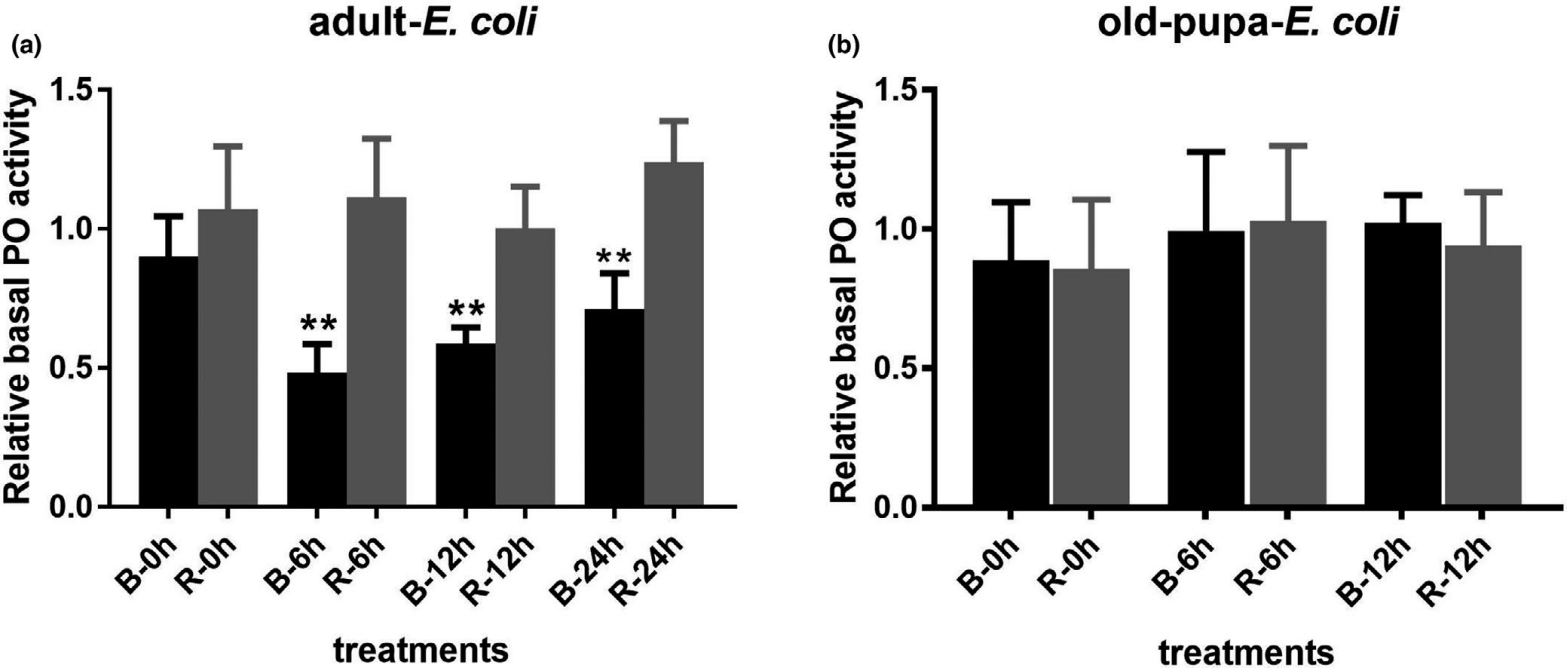
Relative basal PO activity of adults and old pupae in the two color lines of RPW after *Escherichia coli* treatment at different time points. The value of *E. coli* injection groups relative to the mean value of the PBS injection group of two color phenotypes defined as relative PO activity, the unit of PO activity was 1 U (0.001 × △OD_490_/min). ** means significant difference at the level of *p* < .01 by *t* test

There was significant difference of basal PO activity found in adult under natural state (untreated group) (*t*_4_ = 4.38, *p* = .0119). And basal PO activity of red adult was 1.62 times higher than black adult. The relative basal PO activity of adults at different time points after injection treated showed remarkable differences between the two color lines. Higher relative basal PO activity was found in red adults than in black adults at 6 hr (*t*_4_ = 4.638, *p* = .0097), 12 hr (*t*_5_ = 5.124, *p* = .0037), and 24 hr (*t*_5_ = 5.009, *p* = .0041) post *E. coli* injection (Figure 4a). The results with old pupae showed no significant difference between the two phenotypes before treated (*t*_4_ = 0.957, *p* = .3929) and at different time points after treated groups (0 hr: *t*_6_ = 0.201, *p* = .8473; 6 hr: *t*_4_ = 0.1568, *p* = .8830; 12 hr: *t*_5_ = 0.6645, *p* = .5358) (Figure 4b). Basal PO activity of adult was decreased after bacterial or PBS injection, while basal PO activity in pupa showed no significant different.

**FIGURE 5:**
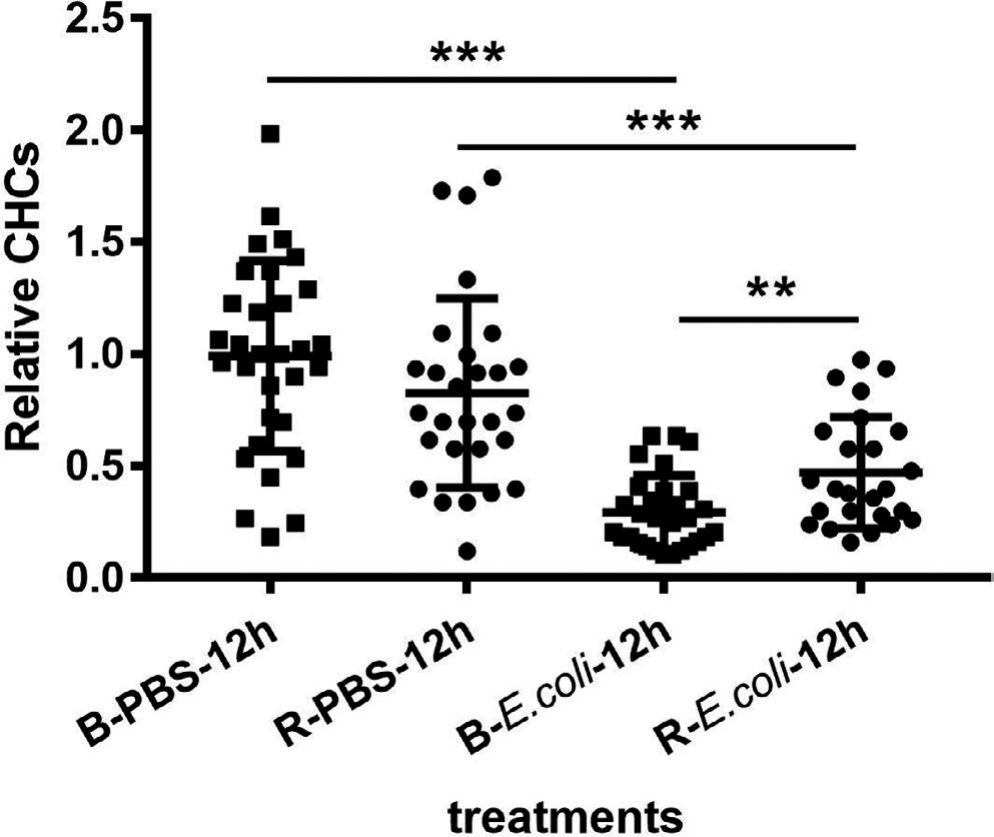
Relative circulating hemocyte counts of adult between the two color lines at 12 hr post injection of PBS or *Escherichia coli*. Relative CHCs was defined as PBS and *E. coli* injection group each relative to the mean value of untreated group, respectively. ** and *** mean significant difference at the levels of *p* < .01 and .001 by *t* test, respectively

Hemocyte density results showed there was no significant difference of CHCs found in adults between two color phenotypes at untreated group (*t*_58_ = 0.919, *p* = .362), while dramatic differences detected in relative CHCs at 12 hr post PBS and *E. coli* injection between the two color lines (red adult: *t*_51_ = −3.681, *p* = .0001; black adult: *t*_58_ = −8.564, *p* < .001). The relative CHCs was significantly higher in red adults than black adults at 12 hr after *E. coli* challenge (*t*_52_ = −3.284, *p* = .002) (Figure 5). However, hemocyte density was extremely low and no significant difference found in old pupae between two color lines in both the noninfection and infection treatments (non‐infection: *t*_46_ = 0.377, *p* = .708; Relative CHCs of 12 hr after PBS and *E. coli* infection: *t*_16_ = 0.003, *p* = .997; *t*_31_ = 1.194, *p* = .241). Therefore, we speculated that in the pupa stage of RPW, hemolymph is not the main agent that produces the immune response.

### 3.4 Relative gene expression in old pupae

**FIGURE 6:**
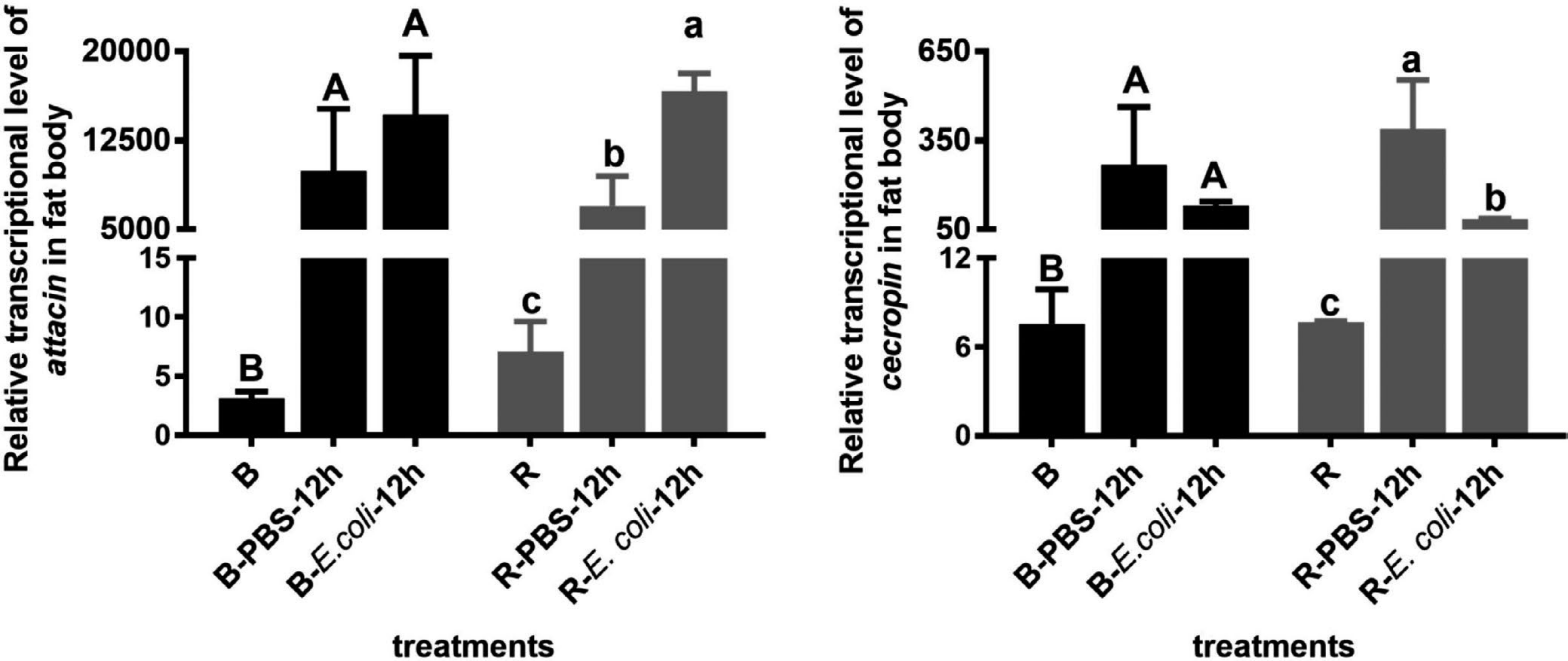
Relative gene expression of antimicrobial peptides in the fat body between the two color lines after *Escherichia coli* challenge. B and R, relative gene expression without injection; B/R‐PBS‐12 hr, relative gene expression at 12 hr post PBS injection; B/R‐*E. coli*‐12 hr, relative gene expression at 12 hr post *E. coli* injection. Different capital letters denote significant difference during the three phases in the black phenotype line at the level of *p* < .05 by ANOVA analyses, and lower case letters denote significant difference in the red phenotype line

The relative expression of two antimicrobial peptides in the fat body in old pupae showed a significant increase in both color phenotypes after the treatments compared with that in untreated pupae (attacin: red *F*_(2,6)_ = 69.94, *p* < .0001, black *F*_(2,6)_ = 9.659, *p* = .0133; cecropin: red *F*_(2,6)_ = 170.336, *p* = .000, black *F*_(2,3.051)_ = 100.376, *p* = .002). However, a significant difference was found only in red pupae between the PBS and *E. coli* injection treatments (attacin: *t*_4_ = −5.592, *p* = .005; cecropin: *t*_4_ = 3.167, *p* = .034) (Figure 6).

**FIGURE 7:**
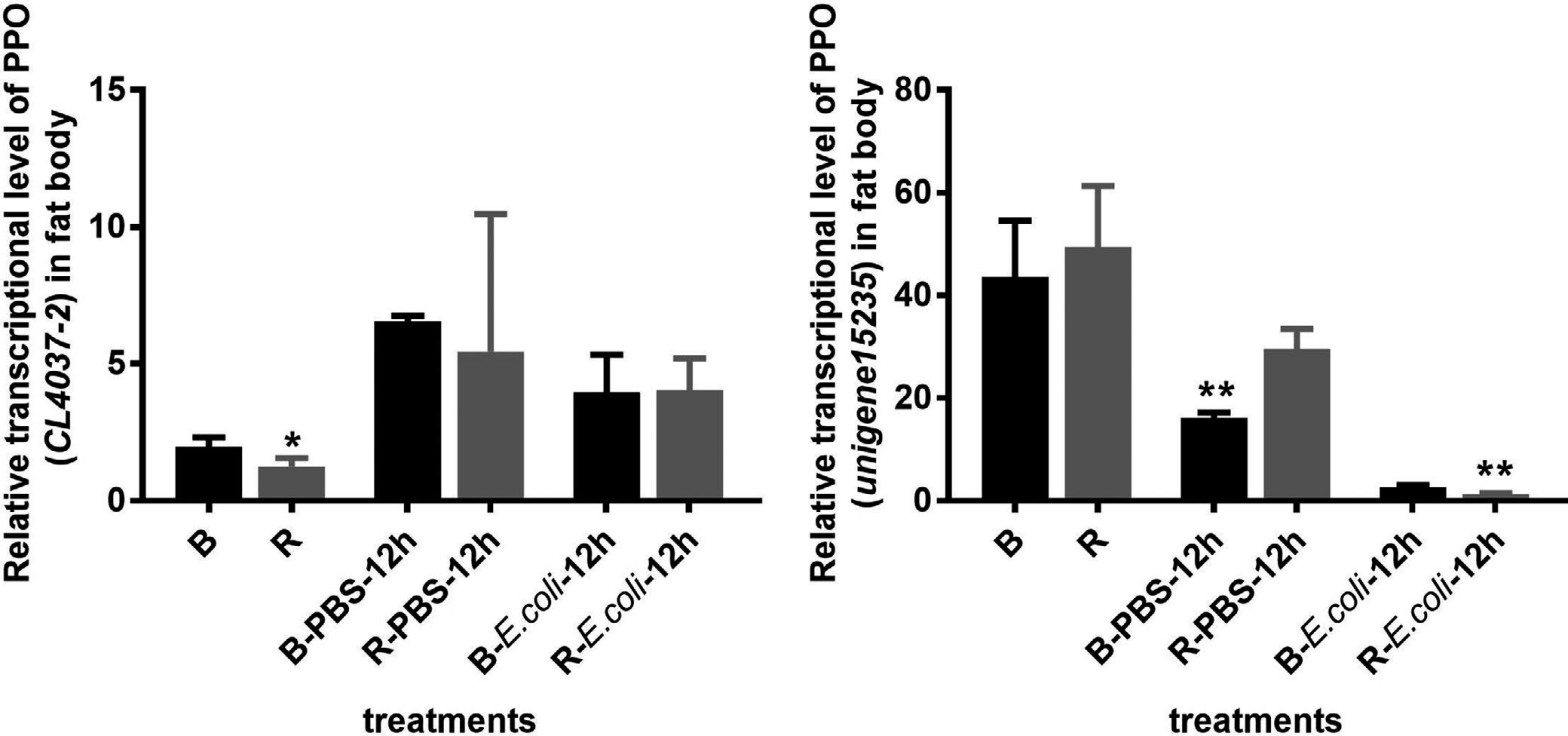
Relative gene expression of PPOs (CL4037‐2 and unigene15235) in the fat body in the two color lines after *Escherichia coli* challenge. B and R, relative gene expression without injection; B/R‐PBS‐12 hr, relative gene expression at 12 hr after PBS injection; B/R‐*E. coli*‐12 hr, relative gene expression at 12 hr after *E. coli* injection. *and ** mean significant difference at the levels of *p* < .05 and .01 by *t* test, respectively

The result of the expression of two PPO genes (*CL4037‐2* and *unigene15235*) in the fat body of old pupae suggested that the relative expression of the *CL4037‐2* gene significantly increased at 12 hr after PBS and *E. coli* injection in black phenotype (*F*_(2,7)_ = 18.35, *p* = .0016), while no significant difference existed between the two phenotypes in bacterial treatment (*t*_5_ = 0.0825, *p* = .938). The relative expression of the unigene*15235* gene declined after treatment, and significant differences were observed between the two phenotypes after bacterial challenge (*t*_6_ = 4.121, *p* = .0062) (Figure 7).

## 4 DISCUSSION

The cuticle structure of RPW was similar to red flour beetle *T. castaneum* (Noh et al., 2016). Cuticle thickness of old pupae with black phenotype was thicker than that with red phenotype, suggesting that the melanism increased the thickness of cuticle in the pigmentation phase. According to the cuticle development process, we speculated that the cuticle tanning process (pigmentation and sclerotization) in black individuals was faster than that in red individuals. The thickness of MESO and ENDO in red individuals rapidly increased from the old‐pupa stage to the adult stage. Results showed that the formation time of MESO and ENDO was different between the two phenotypes. The formation time of these two layers in the red phenotype line was started later than the black phenotype, which may be related to the development processes of epidermis in the two color lines. Transcriptome analysis of pupa epidermis in two color lines showed many genes related to lipid, chitin, and carbohydrate metabolism processes involved in the development of epidermis were upregulated in the red phenotype of RPW (unpublished data). Protein and chitin were the main components of proepidermis including MESO and ENDO (Gillott, 2005; Neville, 1975), which provide the foundation of epidermis formation of the red phenotype of RPW during the pigmentation stage.

Insect cuticular pigmentation is polymorphic, which may link to life history traits such as fecundity, life span, and even immune function. Studies have shown a trade‐off relationship between cuticular pigmentation and fitness characteristics, including immune defense, because pigments and their precursors can be directly used for the immunity response. Similar studies of color phenotype diversity have been demonstrated in many polyphenic insects. Barnes and Siva‐Jothy (2000) proposed that cuticular melanization is an indicator of investment in immunity of mealworm beetle *Tenebrio molitor*. Further studies showed that black *T. molitor* beetles under high density had higher pathogen resistance than tan lines with lower density, which probably resulted from their melanin production (Armitage & Siva‐Jothy, 2005). Wilson et al. (2001) reported that cuticular melanism of nutgrass armyworm *Spodoptera exempta* had a positive relationship with hemocoelic PO. Additional evidence suggested that melanin produced in the cuticle is the result of activation of the PO cascade, which is also important in the humoral immune response of insects (Söderhäll & Cerenius, 1998).

However, the relationship between pigmentation and pathogen resistance is not robust. A study of polymorphic alpine weta *Hemideina maori* suggested that common immune expression was not heightened with dark coloration. The authors noted that the previous finding of a stronger immune response associated with dark coloration in the high‐density melanic phase of polyphonic insects cannot be extended to insects with other forms of discrete color variation (Sandre et al., 2018). A weak relationship was found between cuticular melanism and the fitness of mountain stone weta *Ematurga atomaria*, which does not support a universal trade‐off relationship between body coloration and fitness, suggesting that cuticular biological cost does not necessarily interfere with adaptive evolution (Robb et al., 2003). Reported also revealed that immune challenge had a negative effect on cuticular darkness in the mealworm beetle *T. molitor* (Kangassalo et al., 2016). For the wild population, additional other factors that impact such relationships, such as species‐specific life cycles and developmental, physiological, and environmental factors, should be considered (Bailey, 2011).

The present study revealed a negative relationship between pigmentation, specifically, melanin‐based, and pathogen resistance of two morphs at different life stages. Red adults had significantly higher antimicrobial activity than black adults, suggesting that red adults had a stronger immune response and more antimicrobial substances induced in hemolymph for clearing pathogens. Higher relative PO activity and relative hemocyte counts were detected in red adults than in black adults. Insect melanization is regulated by the prophenoloxidase activity system; PPO is synthesized by hemocytes, and some PPO is transported into the epidermis for cuticular melanization, while some PPO is secreted into the hemolymph for immunity (Ashida & Brey, 1995, Zou et al.,^1995^). On the one hand, there was a trade‐off between melanism and PO in insects. The cuticular melanization in tobacco hornworm *Manduca sexta* had reported that the granular PO, which is distinct from hemolymph PO, is synthesized in the epidermis and transported to the cuticle (Hiruma & Riddiford, 1988). Dark insect required a large amount of granular PO to synthesis melanism, which may lead to the shortage of the necessary amino acids or copper for the manufacture of hemolymph PO (Cotter et al., 2008). The mechanism of melanin trade‐off was similar to the interrelationship between melanin coloration and heavy metals in wasps, common yellowjacket wasps played as an indicator of heavy metal pollution (Skaldina et al., 2020). We speculated that there was a trade‐off between hemolymph PO and granular PO in RPW. A large amount of granular PO required in black phenotype led to less investment in the synthesis of hemolymph PO, which showed a lower intensity of immune response when bacterial challenged. On the other hand, many kinds of prophenoloxidase activating proteinases (PAPs or PPAEs) are involved in PPO activating system, and process in different pathways. Two kinds of PPAEs worked together catalyzing PPO into PO in the scarab beetle *Holotrichia diomphalia* (Kim et al., 2002). PPO in *Manduca sexta* was activated by three PPAFs and two serine proteinase homologs (SPHs) (Jiang et al., 1998). The different pathogen resistance abilities observed between the two differently colored RPW adults may indicate that variant activating factors in the prophenoloxidase‐activating system between two color lines.

Immune response varied with the development stages of insects (Giglio & Giulianini, 2013; Ishaaya & Navon, 1974; Krams et al., 2016). Several studies showed large PO activity differences in different development stages of African Cotton Leafworm *Spodoptera littoralis* (Ishaaya & Navon, 1974). The immune response changed during different development processes with various pigmentation. The level of PO activity in the ground beetle *Carabus (Chaetocarabus) lefebvrei* increased with the degree of pigmentation of the cuticle in larva, pupa, and adult stages (Giglio & Giulianini, 2013). A trade‐off relationship may present among life‐history traits, immune response, and pigmentation. The results for old pupae revealed inconspicuous antimicrobial activity and PO activity than that for RPW adults. Many reasons may result in a variety of immune responses in different development stages. Endocrine factors that regulate molting and metamorphosis of RPW may influence their immune response. Additionally, the immune defense may be traded off against other costly processes that occur during the development stage of old pupae. These kinds of trade‐offs had a closed relationship with their ecological niche. Old pupae cocooned in the hard part of the petiole, which had a well protection, while adults faced more pressure during feeding and oviposition. Adults showed high immune defense to protect themselves, while old pupae may invest more energy for pigmentation and emergence. More hemolymph but much lower hemocyte density was detected in the old‐pupa stage than in the adult stage (old‐pupa: 10^4^–10^5^, adult: 10^6^–10^7^). Therefore, we analyzed the immune response of the fat body in old pupae. The relative expression of two antimicrobial peptides (attacin and cecropin) strikingly increased compared with that in the untreated group at 12 hr after injection in old pupae. After bacterial challenge, only red pupae showed a significant difference, suggesting a stronger immune response in the red phenotype than in the black phenotype during the old‐pupa stage.

The expression of two *PPO* genes changed after foreign organism infection. The relative expression of the *CL4037‐2* increased, but no significant difference was found between the color phenotypes. In contrast, expression of the *unigene15235* decreased, black phenotype showed significantly lower gene expression than red phenotype at 12 hr after PBS injection, while *E. coli* injection showed a reverse result. These results indicated different functions of these two *PPO* genes, and the detailed mechanism requires further study. Similarly, three kinds of PPOs induced by different kinds of hemocytes were found in the fruit fly *Drosophila melanogaster* larvae and played different roles in killing pathogens and parasites (Dudzic et al., 2015).

Pigmentation of insects is not a single and independent process, but a complex process that many factors work together. Insect pigmentation is a pleiotropy phenomenon and shows in various aspects, such as morphology, behavior, and immunity. The results for both adults and old pupae revealed that individuals with a red phenotype had stronger antimicrobial resistance than those with a black phenotype, which is inconsistent with our hypothesis. One possible explanation was that the immune defense in black phenotype was trading off against other costly processes during population development stages. Our other study showed that longer larvae duration and life cycle were found in the black line (unpublished data). This finding showed that it was unilateral to consider only the relationship of the immune response with cuticular pigmentation and that other possible trade‐off factors influencing immune defense, such as population fitness, life cycle, and physiological processes, should be considered for the polyphenic effect of insect cuticular pigmentation.

## CONFLICT OF INTEREST

None declared.

## AUTHOR CONTRIBUTIONS

**Guihua Wang:** Data curation (lead); Formal analysis (lead); Investigation (lead); Writing‐original draft (lead). **Yuxuan Zhou:** Investigation (equal). **Baozhen Tang:** Data curation (equal); Writing‐review & editing (equal). **Habib Ali:** Investigation (equal); Writing‐review & editing (equal). **You‐Ming Hou:** Funding acquisition (lead); Project administration (lead); Supervision (equal); Writing‐review & editing (equal).

## Supporting information

Table S1

## Data Availability

The data that support the findings of this study are available from the corresponding author upon reasonable request.
