# Supplementary material for: Immune function differences between two color morphs of the red palm weevil Rhynchophorus ferrugineus (Coleoptera: Curculionidae) at different life stages
Source: Ecol Evol. 2021 Mar 31;11(10):5702–12. doi: 10.1002/ece3.7474 (PMC8131810; doi:10.1002/ece3.7474)
Supplement: Supplementary file 1 — Table S1 [file ECE3-11-5702-s001.docx]

**Table S1.** qPCR primer sequences.

| Primer name | Sequence |
| --- | --- |
| gapdh-F | CCAAGGGAGCCAAGCAATT |
| gapdh-R | CGCTGATGCCCCTATGTATGT |
| CL4037-2-F | TCGCAACGACAGTCCCTC |
| CL4037-2-R | GACAATAAATCCTATATTCTATATC |
| unigene15235-F | CTCACAGTTACCGCAACGCTAGTCC |
| unigene15235-R | CGTTCACGCTTGTTAAAG |
| attacin-F | TGGTTCTGGTGCCCAAGTGA |
| attacin-R | GCCATAACGATTCTTGTTGGAGTA |
| cecropin-F | CAGAAGCTGGTTGGTTGAAGA |
| cecropin-R | GCAACACCGACATAACCCTGA |
